# Effect of a pre-exercise energy drink (Redline®) on muscular endurance of the trunk

**DOI:** 10.1186/1550-2783-8-S1-P13

**Published:** 2011-11-07

**Authors:** David Temple, Jay Dawes, Liette Ocker, Frank Spaniol, Donald Melrose, Allison Murray

**Affiliations:** 1 Department of Kinesiology. Texas A & M University-Corpus Christi, Corpus Christi, TX, USA

## Background

Muscular endurance of the trunk is associated with successful performance in athletics, as well as activities of daily living. Furthermore, muscular endurance of the trunk may also play a critical role in injury prevention by allowing individuals to better withstand the effects of repetitive stressors. Pre-exercise, high energy supplements are frequently consumed as a method of improving exercise performance during an acute bout of exercise. Thus, the use of such supplements prior to an exercise session may allow the lifter to perform a greater total volume of work during training sessions. The purpose of this study was to investigate the effect of a high energy liquid supplement on a muscular endurance exerciseof the trunk.

## Methods

Forty-one (n=41) healthy males (21.73 ± 1.74 yrs; 176.48 ± 7.54 cm; 81.16 ± 10.94 kg) volunteered to participate in this study. All test subjects completed a health history and caffeine usage questionnaires, as well as a consent form prior to participation. Subjects completed a pre and post sit-up to fatigue test within a week of one another. During the post-test session subjects were either given four ounces of an energy supplement (Redline by VPX) or a placebo, 30 minutes prior to testing. Administration of the supplement was double blind. Twenty-three (n=23) subjects received the supplement, while eighteen (n=18) subjects received the placebo. A 2 x 2 factorial ANOVA was used to determine between group differences for the muscular endurance assessments,at an alpha level of 0.10.

## Results

Analysis of the data revealed a significant interaction at the alpha 0.10 level, *F* (1, 40) = 2.79, *p* = 0.075. As indicated, the degrees of freedom are limited by sample size; therefore, with more subjects in both the treatment and placebo group the expected outcome would be magnified. However, further examination of the data revealed an important finding, the sit-up scores of the treatment group were significantly higher for the posttest (59.00 ± 20.65) than the sit-up scores for the placebo group (53.06 ± 20.63). The treatment effect was further emphasized when comparing pretest sit-up scores. There was no significant difference in pretest sit-up scores between the groups (treatment: 52.13 ± 18.94, placebo: 53.44 ± 17.73), however posttest scores revealed significantly higher scores in the treatment group (13.2%) when compared to the placebo (- 0.7%).

## Conclusions

The results of this study indicate thatthe pre-exercise liquid energy supplement investigated had a significant effect on upper-body muscular endurance as measured by the sit-up to fatigue test when taken within 30 minutes of the exercise bout.

